# Fisetin Protects PC12 Cells from Tunicamycin-Mediated Cell Death via Reactive Oxygen Species Scavenging and Modulation of Nrf2-Driven Gene Expression, SIRT1 and MAPK Signaling in PC12 Cells

**DOI:** 10.3390/ijms18040852

**Published:** 2017-04-17

**Authors:** Jui-Hung Yen, Pei-Shan Wu, Shu-Fen Chen, Ming-Jiuan Wu

**Affiliations:** 1 Department of Molecular Biology and Human Genetics, Tzu Chi University, Hualien 970, Taiwan; imyenjh@hotmail.com; 2 Department of Biotechnology, Chia Nan University of Pharmacy and Science, Tainan 717, Taiwan; dc7575@gmail.com; 3 Department of Health and Nutrition, Chia Nan University of Pharmacy and Science, Tainan 717, Taiwan; ichensf@mail.cnu.edu.tw

**Keywords:** fisetin, tunicamycin, HO-1, p38 MAPK, SIRT1

## Abstract

Background: Fisetin (3,7,3′,4′-tetrahydroxyflavone) is a dietary flavonol and exhibits antioxidant, anti-inflammatory, and neuroprotective activities. However, high concentration of fisetin is reported to produce reactive oxygen species (ROS), induce endoplasmic reticulum (ER) stress and cause cytotoxicity in cancer cells. The aim of this study is to investigate the cytoprotective effects of low concentration of fisetin against tunicamycin (Tm)-mediated cytotoxicity in neuronal-like catecholaminergic PC12 cells. Methods: Cell viability was assayed by MTT (3-(4,5-dimethylthiazol-2-yl)-2,5-diphenyltetrazolium bromide) and apoptotic and autophagic markers were analyzed by Western blot. Gene expression of unfolded protein response (UPR) and Phase II enzymes was further investigated using RT-Q-PCR or Western blotting. Intracellular ROS level was measured using 2′,7′-dichlorodihydrofluorescein diacetate (H_2_DCFDA) by a fluorometer. The effects of fisetin on mitogen activated protein kinases (MAPKs) and SIRT1 (Sirtuin 1) signaling pathways were examined using Western blotting and specific inhibitors. Results: Fisetin (<20 µM) restored cell viability and repressed apoptosis, autophagy and ROS production in Tm-treated cells. Fisetin attenuated Tm-mediated expression of ER stress genes, such as glucose-regulated proteins 78 (GRP78), C/EBP homologous protein (CHOP also known as GADD153) and Tribbles homolog 3 (TRB3), but induced the expression of nuclear E2 related factor (Nrf)2-targeted heme oxygenase (HO)-1, glutamate cysteine ligase (GCL) and cystine/glutamate transporter (xCT/SLC7A11), in both the presence and absence of Tm. Moreover, fisetin enhanced phosphorylation of ERK (extracellular signal-regulated kinase), JNK (c-JUN NH_2_-terminal protein kinase), and p38 MAPK. Addition of JNK and p38 MAPK inhibitor significantly antagonized its cytoprotective activity and modulatory effects on UPR. Fisetin also restored Tm-inhibited SIRT1 expression and addition of sirtinol (SIRT1 activation inhibitor) significantly blocked fisetin-mediated cytoprotection. In conclusion, this result shows that fisetin activates Nrf2, MAPK and SIRT1, which may elicit adaptive cellular stress response pathways so as to protect cells from Tm-induced cytotoxicity.

## 1. Introduction

Endoplasmic reticulum (ER) is a dynamic organelle responsible for the synthesis, correct folding, post-translational modification and transport of secretory proteins. Disturbance of ER function leads to the accumulation of unfolded or misfolded proteins in the ER lumen, and is referred to as ER stress [1]. The correlations between ER stress and neuronal degenerative diseases, including Alzheimer’s (AD), Parkinson’s (PD) and amyotrophic lateral sclerosis (ALS), are well documented [2]. ER stress can also be chemically induced by tunicamycin (Tm), an antibiotic produced by *Streptomyces lysosuperificus.* Tm induces ER stress by inhibiting microsomal enzyme *N*-acetylglucosamine-1-phosphate transferase, which is responsible for the glycosylation of newly synthesized proteins [3].

The unfolded protein response (UPR) is a series of events that cells activate in order to cope with ER stress and to re-establish ER homeostasis [4,5]. UPR contains three parallel signalling branches: IRE1 (inositol-requiringprotein1)-XBP1 (X-box-binding protein 1), ATF6 (activating transcription factor 6) and PERK (protein kinase RNA-like ER kinase)-eIF2α (eukaryotic translation initiation factor 2α)-ATF4 (activating transcription factor 4). The three transcription factors activated, XBP1, ATF6, and ATF4, individually and collectively upregulate the transcription of ER chaperones, such as glucose-regulated proteins 78 (GRP78), ER biosynthetic machinery, and ER associated degradation (ERAD) components in order to resolve ER stress [6]. In the case of chronic or unmitigated ER stress, cell death pathways are induced [7]. ER stress-induced apoptosis is mediated mainly by C/EBP homologous protein (CHOP/GADD153) [8,9].

Emerging data also suggest that, in addition to UPR, autophagy is part of the global ER stress response [10,11]. Autophagy is an evolutionarily conserved mechanism that sequesters undesired cellular material into autophagosomes for delivery to lysosomes for degradation [12]. Autophagosome formation is controlled by a well-orchestrated action of a distinctive set of autophagy-related (Atg) proteins. A key reaction involves the lipidation of Atg8 in autophagic membranes, which is facilitated by the Atg12–Atg5 conjugate [13].

Flavonoids are a group of bioactive compounds that are commonly found in fruits, vegetables, nuts and beans. Flavonoids and their metabolites can transport across the blood–brain barrier (BBB), making them ideal targets in the therapeutic utility for neurodegenerative disorders [14]. Numerous studies indicate that the anti-neurodegeneration effects of flavonoids are consistent with their roles as activators of mitogen activated protein kinases (MAPKs), SIRT1 (Sirtuin 1) or Nrf2 (nuclear E2 related factor 2)-ARE (antioxidant response element) [15,16,17,18]. The MAPK family, including ERK (extracellular signal-regulated kinase), JNK (c-JUN NH_2_-terminal protein kinase), and p38, plays an essential role in transduction extracellular signals to cellular response via a cascade of phosphorylation events [19]. SIRT1 is an NAD^+^-dependent class III histone deacetylase, which regulates cellular metabolism, stress resistance, cellular survival, cellular senescence/aging, and inflammation-immune function [20]. It has been shown that SIRT1 overexpression in neurons promotes neurite outgrowth and cell survival [21]. Recently, it has been reported that the combination of tyrosol, a natural antioxidant phenol, and *S*-adenosylmethionine increased SIRT1 protein and its nuclear relocalization in ethanol-treated HepG2 cells and protected cells from oxidative stress [22]. Nrf2-ARE is a primary sensor and oxidative stress regulator [23]. Nrf2 activation upregulates the expression of a group of functionally diverse cytoprotective proteins, such as NAD(P)H, NAD(P)H:quinone oxidoreductase 1 (NQO1), superoxide dismutase (SOD), glutathione *S*-transferase (GST), glutathione peroxidase (GPx), heme oxygenase-1 (HO-1), glutamate-cysteine ligase (GCL), catalase, and thioredoxin [16].

**Figure 1:**
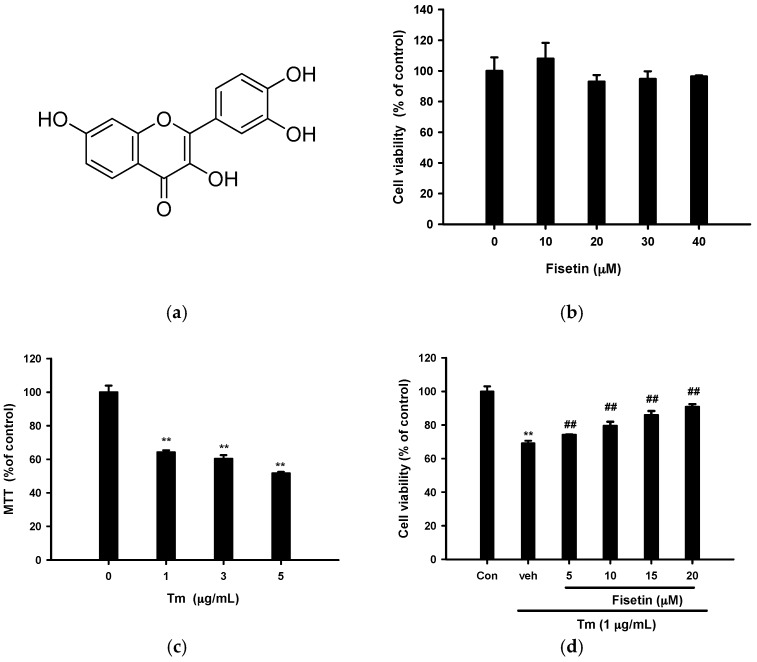
Effects of fisetin on tunicamycin (Tm)-induced PC12 cell death. (**a**) Chemical structure of fisetin; (**b**) Fisetin alone does not change the cell viability of PC12 cells; (**c**) Tm causes cell death in PC12 cells; (**d**) Fisetin inhibits Tm-mediated cytotoxicity in PC12 cells. Cells were treated with the indicated concentration of compound or vehicle control (0.1% dimethyl sulfoxide, DMSO) for 30 min followed by exposure to Tm for an additional 16 h at 37 °C. Cell viability was measured by MTT (3-(4,5-dimethylthiazol-2-yl)-2,5-diphenyltetrazolium bromide), as described in Materials and Methods. ** *p* < 0.01 represents significant differences compared with vehicle control (Con, without Tm). ## *p* < 0.01 represents significant differences compared with the Tm-treated vehicle (veh).

Fisetin (3,7,3′,4′-tetrahydroxyflavone, Figure 1a) is a flavonol with a wide range of bioactivites, including antioxidant, anti-inflammatory, anti-cancer and neuroprotective effects. It has been reported that fisetin protects rat pheochromocytoma PC12 cells from MPP^+^-induced toxicity by upregulation of the expression of Nrf2-induced antioxidant enzymes [24]. Fisetin has been shown to promote neuronal differentiation and long-term potentiation, maintain cognitive function and enhance memory in an animal model [25,26,27]. Fisetin also protects PC12 cells from hypoxia-induced PC12 cell death via activating the HIF1α, MAPK and PI3K/Akt signaling pathways [28]. Taken together, these studies show that fisetin is a potential compound with neuroprotective activity. However, the cytoprotective effect of fisetin on neuronal cells exposed to ER stress inducer is still unknown. The aim of this study is to dissect the molecular and biochemical pathways involved in fisetin-mediated cytoprotection in Tm-treated PC12 cells.

## 2. Results

### 2.1. Fisetin Protects PC12 Cells from Tm-Mediated Cell Death

It was reported that fisetin at low concentrations provides neuroprotection in several models [25,26,27,28,29], while at high concentrations it induces reactive oxygen species (ROS) production, ER stress and cell death in some tumor cells [30,31,32]. We found that treatment of PC12 cells with fisetin (up to 40 µM) alone for 16 h did not alter cell viability (Figure 1b). It is known that Tm blocks *N*-glycosylation of proteins, thus leading to ER stress [3] and ultimately apoptosis in PC12 cells [33,34]. Figure 1c shows that Tm (1–5 µg/mL) caused 30–40% PC12 cell death after 16 h. Treatment of PC12 cells with fisetin (5–20 µM) dose-dependently reversed 1 µg/mL Tm-mediated cell death (Figure 1d). The cytoprotective effects of fisetin were reconfirmed by Calcein AM viability dye staining (Figure S1) [35].

### 2.2. Fisetin Inhibits Tm-Mediated Apoptotic and Autophagic Marker Protein Expression

**Figure 2:**
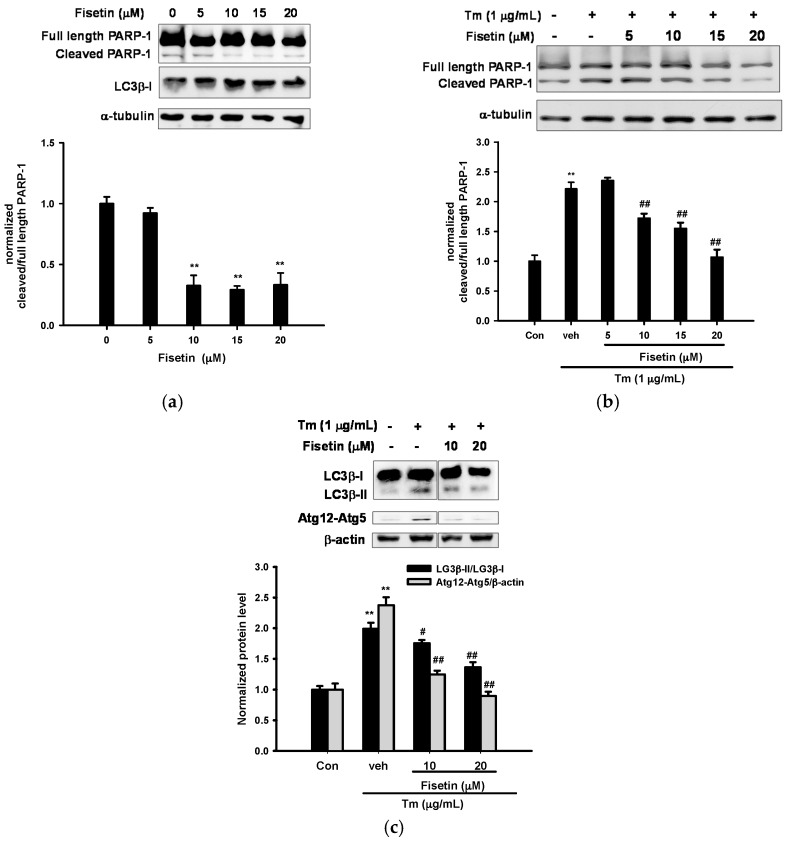
Effect of fisetin on apoptotic and autophagic marker protein expression. (**a**) Effect of fisetin alone on poly (ADP-ribose) polymerase-1 (PARP-1) activation and microtubule-associated protein 1 light chain 3 (LC3) conversion; (**b**) Effect of fisetin on Tm-activated PARP-1 activation; (**c**) Effect of fisetin on Tm-mediated conversion of LC3B and formation of Atg12–Atg5 conjugate. Cells were treated with the indicated reagent for 16 h and cell lysates were prepared and subjected to immunoblotting. The blots are representative from one of three independent experiments. Data obtained from immunoblots were then analyzed using ImageJ software. Data represent the mean ± SD of three independent experiments. (**a**) ** *p* < 0.01 represents significant differences compared with no fisetin control; (**b**,**c**) ** *p* < 0.01 represents significant differences compared with vehicle control (without Tm). # *p* < 0.05; ## *p* < 0.01 represent significant differences compared with the Tm-treated vehicle group.

Poly (ADP-ribose) polymerase-1 (PARP-1) is specifically proteolysed by caspases to a 24 and a 89 kDa fragment during the execution of the apoptotic program [36]. Figure 2a shows that treatment of PC12 cells with fisetin (10–20 µM) in the absence of Tm for 16 h decreased PARP-1 processing in a dose-dependent manner, although it did not significantly change cell viability (Figure 1b). Tm (1 µg/mL) treatment increased PARP-1 cleavage as compared with no Tm control, and co-treatment with fisetin counteracted this reaction (Figure 2b). Band quantification shows that Tm induced a significant increase in the ratio of cleaved/full length PARP-1, and fisetin (10‒20 µM) dose-dependently attenuated Tm-mediated cleavage. This result indicates that fisetin serves as an anti-apoptotic agent in PC12 cells.

Autophagy and apoptosis are important and interconnected stress response mechanisms. Microtubule-associated protein 1 light chain 3 (LC3) is the mammalian ortholog of yeast Atg8, and is required for the formation of autophagosome membrane. The conversion of LC3β from LC3β-I (free form, 18 kDa) to LC3β-II (phosphatidylethanolamine-conjugated form, 16 kDa) is an initiating step in autophagy in mammals [37]. PC12 cells cultured in the absence of Tm did not cause the conversion of LC3β (Figure 2a). In comparison, Figure 2c shows that an increase in the LC3β-II in PC12 cells was observed in the presence of Tm (1 µg/mL), confirming that autophagy was induced by Tm. Co-treatment of cells with 10 and 20 µM fisetin dose-dependently reduced Tm-mediated increase in the ratio of LC3β-II/LC3β-I. The formation of Atg12–Atg5 conjugate is a key factor in regulating the formation of autophagosome [38]. In parallel to the results found for LC3β conversion, Tm treatment for 16 h also enhanced Atg12–Atg5 conjugate formation and co-treatment of fisetin (10 and 20 µM) blocked its formation. This result suggests that fisetin represses Tm-mediated autophagy in PC12 cells.

### 2.3. Fisetin Inhibits Tm-Mediated Endoplasmic Reticulum (ER) Stress Gene Expression

**Figure 3:**
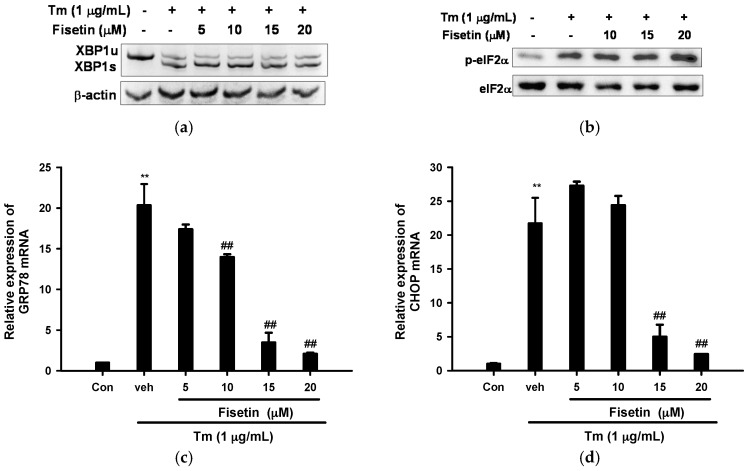

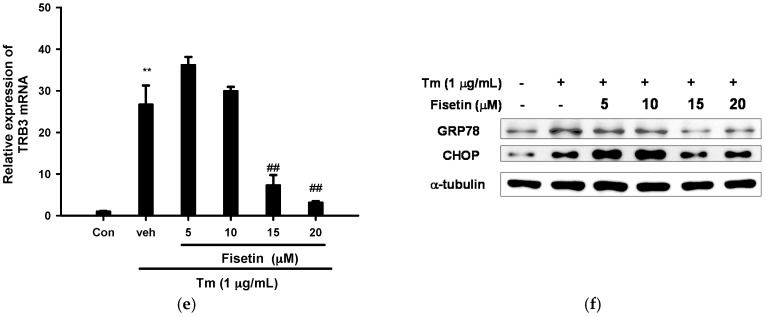
Effect of fisetin on Tm-mediated unfolded protein response (UPR). (**a**,**c**–**e**) Cells were treated with the indicated reagent for 6 h at 37 °C and RNA was prepared. Semi-quantitative RT-PCR and polyacrylamide electrophoresis was employed for the analysis of mRNA levels of XBP1s and XBP1u. RT-Q-PCR was used for the analysis of mRNA levels of glucose-regulated proteins 78 (GRP78), C/EBP homologous protein (CHOP) and tribbles-related protein 3 (TRB3); (**b**) Cells were treated with the indicated reagent for 4 h and levels of phospho-eIF2α and eIF2α were analyzed using immunoblotting; (**f**) Cells were treated with the indicated reagent for 8 h and GRP78 and CHOP in cell lysates were analyzed using immunoblotting. Data represent the mean ± SD of three independent experiments. ** *p* < 0.01 represents significant differences compared with vehicle control (without Tm). ## *p* < 0.01 represents significant differences compared with the Tm-treated vehicle group.

We further investigated the effect of fisetin on Tm-mediated ER stress response. The first target was X-box-binding protein 1 (XBP1) mRNA splicing, a critical signal transducer in the unfolded protein response. The levels of the unspliced XBP1 (XBP1u) and active spliced XBP1 (XBP1s) mRNA were measured by RT-PCR and subsequent polyacrylamide electrophoresis. It was found that Tm (1 µg/mL) treatment significantly increased XBP1 splicing, but co-treatment with fisetin (5–20 µM) did not change the relative level of XBP1s to that of XBP1u (Figure 3a). A similar phenomenon was also observed for eIF2α phosphorylation, another ER stress signal transducer upstream of ATF4. Tm (1 µg/mL) treatment markedly induced eIF2α phosphorylation, while fisetin (10–20 µM) did not change its level (Figure 3b). These results indicate that fisetin did not affect Tm-activated IRE1-XBP1 or PERK-eIF2 pathway.

We then investigated whether fisetin affected Tm-mediated ER-target gene expression. GRP78 and CHOP are canonically upregulated during apoptosis induced by ER stress. It was found that Tm treatment caused a strong increase in GRP78 mRNA expression, up by ~20-fold, while co-treatment with fisetin (10–20 µM) dose-dependently reduced its expression and 90% inhibition was noted at 20 µM (Figure 3c).

Figure 3d shows that Tm induced CHOP mRNA by ~22-fold and co-treatment with 15 and 20 µM fisetin could dose-dependently attenuate the upregulation by 77% and 88%, respectively. It has been reported that tribbles-related protein 3 (TRB3), a target gene of CHOP, was responsible for Tm-mediated PC12 apoptosis [39]. In parallel to the results found for CHOP mRNA expression, Figure 3e shows that treatment of PC12 cells with 1 µg/mL Tm increased TRB3 mRNA expression by 24.7-fold, and 15 and 20 µM fisetin could dose-dependently attenuate its upregulation. In conclusion, fisetin (10–20 µM) inhibited Tm-upregulated UPR mRNA expression in a dose-dependent manner.

Western blotting reveals that treatment of PC12 cells with Tm modestly increased GRP78 and CHOP protein expression. Fisetin dose-dependently attenuated GRP78 expression; while its effect on Tm-mediated CHOP protein expression was less prominent (Figure 3f). The less obvious inhibition on CHOP protein expression could be due to the poor detection sensitivity and specificity of antibodies against rat CHOP.

### 2.4. Fisetin Scavenges Reactive Oxygen Species (ROS) Production

**Figure 4:**
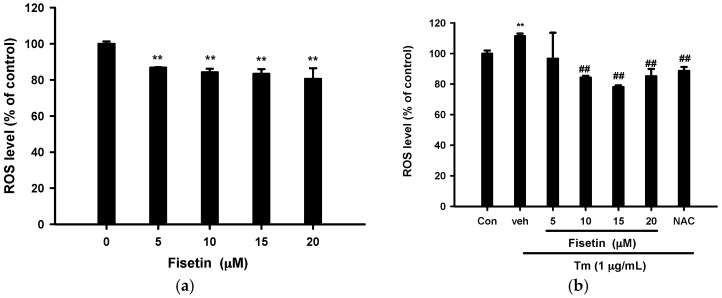
Effects of fisetin on intracellular ROS production. (**a**) PC12 cells were treated with fisetin for 16 h at 37 °C; (**b**) PC12 cells were treated with fisetin 30 min prior to Tm insult for 16 h at 37 °C. Intracellular ROS production was measured by 2′,7′-dichlorodihydrofluorescein diacetate (H_2_DCFDA). Data represent the mean ± SD of three independent experiments. (**a**) ** *p* < 0.01 represents significant differences compared with no fisetin control; (**b**) ** *p* < 0.01 represents significant differences compared with control. ## *p* < 0.01 represents significant differences compared with the Tm-treated vehicle group.

It has been reported that Tm induces apoptosis in leukemia U937 cells through ROS generation [40]. Figure 4a shows that treatment of PC12 cells with fisetin (5–20 µM) alone for 16 h could decrease ROS level by about 15–20%. Treatment of PC12 cells with Tm (1 µg/mL) for 16 h modestly increased intracellular ROS production by about 11% (Figure 4b). Fisetin (10–20 µM) and *N*-acetyl-cysteine (NAC) (1 mM), a well-known antioxidant, completely abolished Tm-mediated ROS overproduction to a level lower than that of vehicle control. Current data support the notion that fisetin serves as a ROS scavenger in PC12 cells [28].

### 2.5. Fisetin Induces Nrf2-Driven Oxidative Stress Response Gene Expression

**Figure 5:**
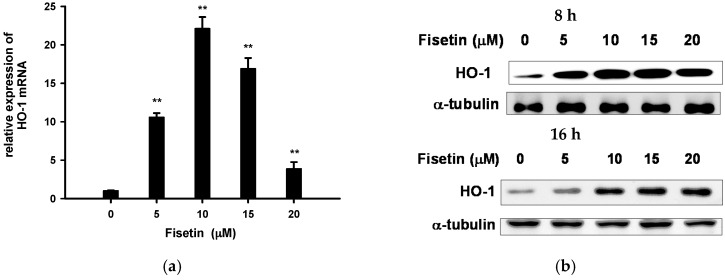

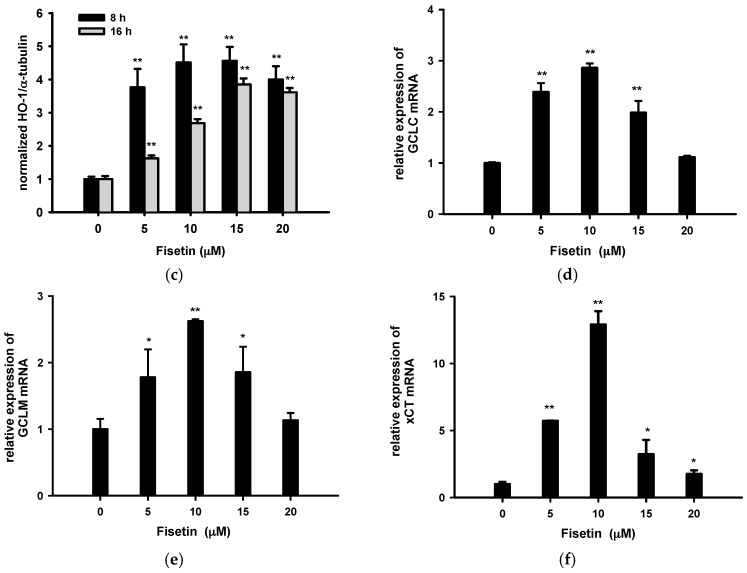
Fisetin induces Nrf2-targeted gene expression in the absence of Tm. (**a**,**d**–**f**) PC12 cells were treated with 5–20 µM Fisetin for 6 h. RNA was prepared and the mRNA levels of HO-1, GCLC, GCLM and xCT were analyzed by RT-Q-PCR and normalized to β-actin; (**b**,**c**) PC12 cells were treated with 5–20 µM fisetin for 8 or 16 h; subsequently, the cell lysates were subjected to immunoblotting for HO-1 and α-tubulin. Immunoblots were then analyzed using ImageJ software. Data represent the mean ± SD of three independent experiments. * *p* < 0.05, and ** *p* < 0.01 represents significant differences compared with vehicle control.

Nrf2 (nuclear E2 related factor 2)-ARE (antioxidant response element) is a primary sensor and oxidative stress regulator [23]. Fisetin (10 µM) was reported to cause activation of Nrf2-ARE and upregulation of heme oxygenase-1 (HO-1), cystine/glutamate transporter (xCT/SLC7A11) and glutamate cysteine ligase (GCL) in HT22 cells [25,41]. Figure 5a shows that fisetin (5–20 µM) treatment for 6 h significantly induced HO-1 mRNA expression in an inverted U curve, and the greatest increase (22-fold) was found for 10 µM. Consequently, HO-1 protein expression was significantly stimulated after fisetin treatment (Figure 5b). Band quantification shows that a hormetic dose–response curve was noted after 8 h treatment and ~4.5-fold induction was caused by 10 and 15 µM fisetin (Figure 5c). Treatment with fisetin for 16 h induced less HO-1 protein expression than treatment for 8 h, and plateau induction (~3.7-fold) was reached by doses equal to or higher than 15 µM.

Parallel induction effects were also observed for Nrf2-driven mRNA expression of glutathione metabolism related genes, such as GCLC (catalytic subunit of GCL), GCLM (modifier subunit of GCL) and xCT (Figure 5d–f). These results suggest that fisetin induced a hormetic effect on Nrf2-driven mRNA expression of Phase II antioxidant enzymes.

**Figure 6:**
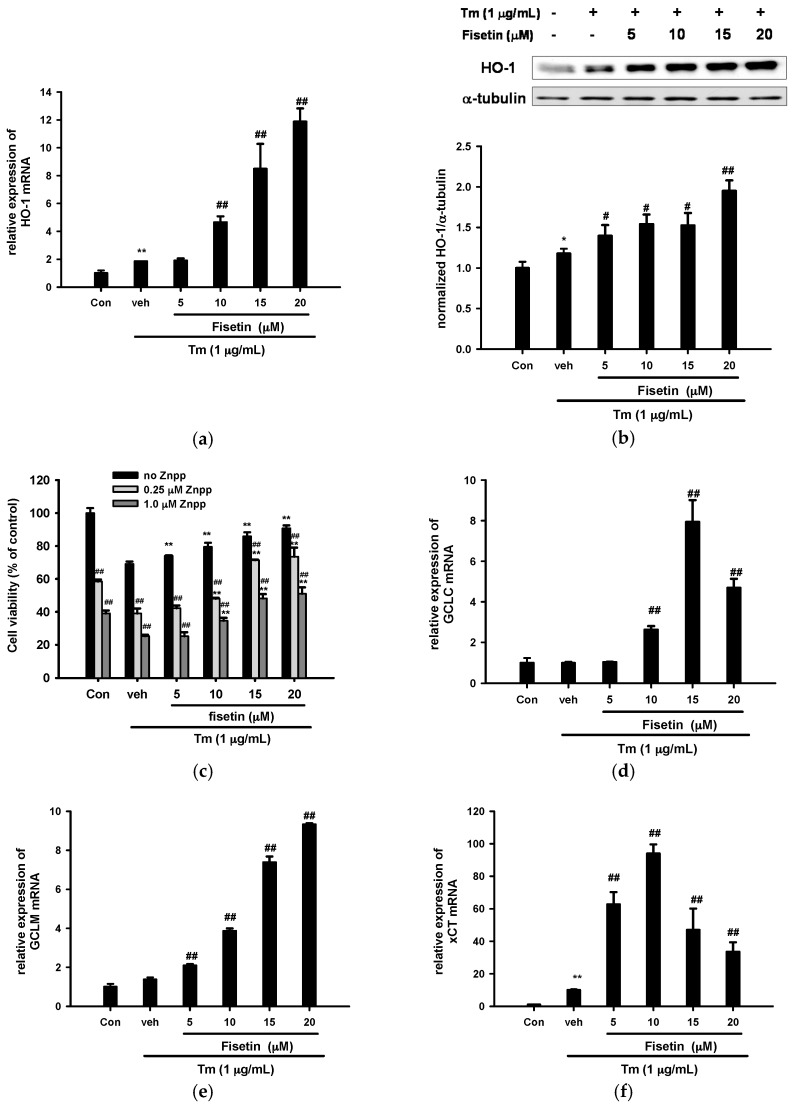
Effects of fisetin on Nrf2-targeted gene expression in the presence of Tm. (**a**,**d**–**f**) PC12 cells were treated with fisetin 30 min prior to Tm co-treatment for 12 h at 37 °C. The mRNA levels of HO-1, GCLC, GCLM and xCT were analyzed by RT-Q-PCR and normalized to β-actin; (**b**) PC12 cells were treated with fisetin 30 min prior to Tm co-treatment for 16 h. Immunoblotting of HO-1 and α-tubulin were employed and the bands were quantitated using ImageJ. The data represent the mean ± SD of three independent experiments. ** *p* < 0.01 represents significant differences compared with vehicle control (without Tm); # *p* < 0.01, and ## *p* < 0.01 represent significant differences compared with the Tm-treated vehicle group; (**c**) Effects of inhibition of HO-1 activity on cell viability. PC12 cells were pretreated for 30 min with Znpp, and fisetin (5–20 µM) was then added 30 min prior to Tm exposure for 16 h. MTT was used to analyze the cell viability. Data represent the mean ± SD of three independent experiments. ** *p* < 0.01 represents significant differences compared with the Tm-treated respective vehicle group. ## *p* < 0.01 represents significant differences compared with the respective no inhibitor group.

We further investigated how fisetin affected Nrf2-driven gene expression in the presence of Tm. Figure 6a shows that Tm (1 µg/mL) only slightly stimulated HO-1 mRNA expression by 1.84-fold, and co-treatment with fisetin (10–20 µM) enhanced HO-1 upregulation dose-dependently. Figure 6b shows that in parallel to the results observed in mRNA expression, treatment of PC12 cells with Tm (1 µg/mL) for 16 h slightly upregulated HO-1 protein expression and fisetin enhanced HO-1 protein expression dose-dependently.

To further investigate whether the increased survival rate seen in fisetin-treated cells was dependent on HO-1 activity, PC12 cells were treated with ZnPP (zinc protoporphyrin IX), a potent competitive inhibitor of HO enzyme activity, for 30 min, followed by various concentrations of fisetin for 30 min before exposure to Tm (1 µg/mL) for 16 h. Figure 6c shows that the addition of ZnPP (0.25 and 1 µM) caused cytotoxicity on PC12 cells in all groups, and attenuated the fisetin-mediated cytoprotective effect dose-dependently. These results indicate that HO-1 activity may provide a survival advantage in PC12 cells.

We then turned to investigate how Tm affects GSH-related gene expression. The expressions of GCLC and GCLM were not affected by Tm (1 µg/mL) (Figure 6d,e). Fisetin (5–20 µM) induced GCLM expression dose-dependently (Figure 6e), while GCLC expression was impacted following a hormetic dose–response curve (Figure 6d).

It was reported that xCT expression was upregulated by both Nrf2 and ATF4 [42]. We found that, different from other Nrf2-driven genes, Tm strongly stimulated xCT expression by 10-fold. In parallel to the results observed in the absence of Tm, fisetin (5–20 µM) enhanced xCT overexpression in an inverted U response curve in the presence of Tm (Figure 6f).

### 2.6. Fisetin Activates Mitogen-Activated Protein Kinase (MAPK) Signaling Pathways

**Figure 7:**
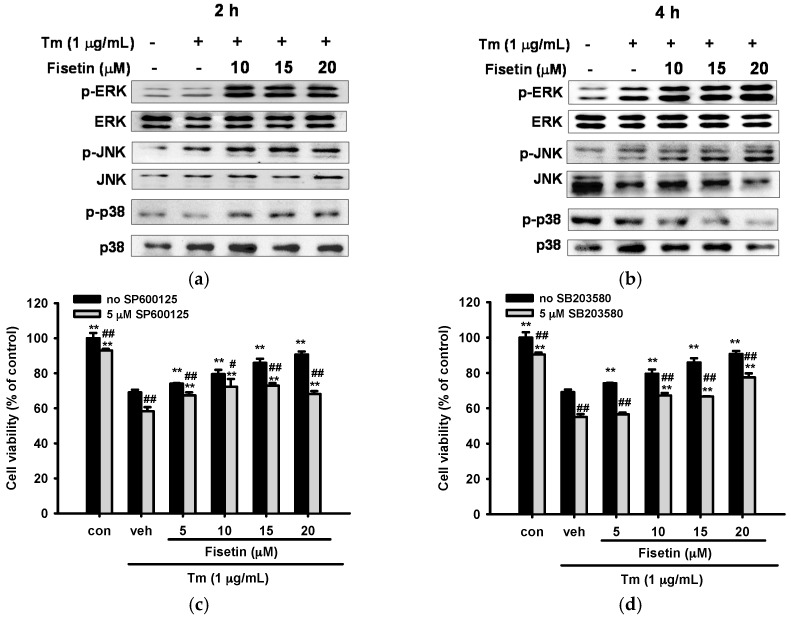
Involvement of MAPK signaling pathways in the cytoprotective effects of fisetin. (**a**,**b**) Effects of fisetin on MAPK activation. PC12 cells were treated with fisetin 30 min prior to Tm (1 µg/mL) treatment for 2 and 4 h at 37 °C. Cell lysates were prepared and the levels of MAPK activation were analyzed by immunoblotting; (**c**,**d**) Effects of inhibition of JNK and p38 MAPK activation on cell viability. PC12 cells were pretreated for 30 min with 5 µM JNK (SP600125) or p38 MAPK inhibitor (SB203580) and 5–20 µM fisetin was then added 30 min prior to Tm (1 µg/mL) exposure for 16 h. MTT was used to analyze the cell viability. Data represent the mean ± SD of three independent experiments. ** *p* < 0.01 represents significant differences compared with the Tm-treated respective vehicle group. # *p* < 0.05 and ## *p* < 0.01 represent significant differences compared with the respective no inhibitor group.

Induction of Nrf2 by flavonoids has been associated with the activation of various members of the MAPK family [43]. To investigate how fisetin affects MAPK activation, PC12 cells were treated with 10, 15 and 20 μM fisetin for 30 min prior to Tm (1 µg/mL) exposure for 2 and 4 h, and cell lysates were immunoblotted with specific antibodies. Figure 7a,b show that treatment of 1 µg/mL Tm for 2 and 4 h induced ERK and JNK activation, but not p38 MAPK activation. Co-treatment with fisetin (10–20 µM) significantly augmented ERK, JNK and p38 MAPK activation with different patterns: enhancement of ERK and JNK activation was noted during the test period, while p38 MAPK phosphorylation was only found 2 h after Tm insult.

To study the cytoprotective role of the MAPK pathways, PC12 cells were pre-incubated for 30 min with 5 µM inhibitor for each pathway, U0126 (ERK), SP600125 (JNK) and SB203580 (p38 MAPK), and 5–20 µM fisetin was then added 30 min prior to Tm (1 µM) exposure for 16 h. Figure 7c,d show that addition of JNK inhibitor SP600125 and p38 MAPK specific inhibitor SB203580 significantly exacerbated Tm-mediated cytotoxicity and attenuated the cytoprotective effects of fisetin. On the other hand, MEK inhibitor U0126 did not exert any effect on cell viability in the presence of Tm (Figure S2). These results indicate that JNK and p38 MAPK signaling pathways, but not ERK, may be associated with the cytoprotective effect of fisetin.

### 2.7. Contribution of JNK and p38 MAPK Pathways to ER Stress and Nrf2-Driven Gene Expression

**Figure 8:**
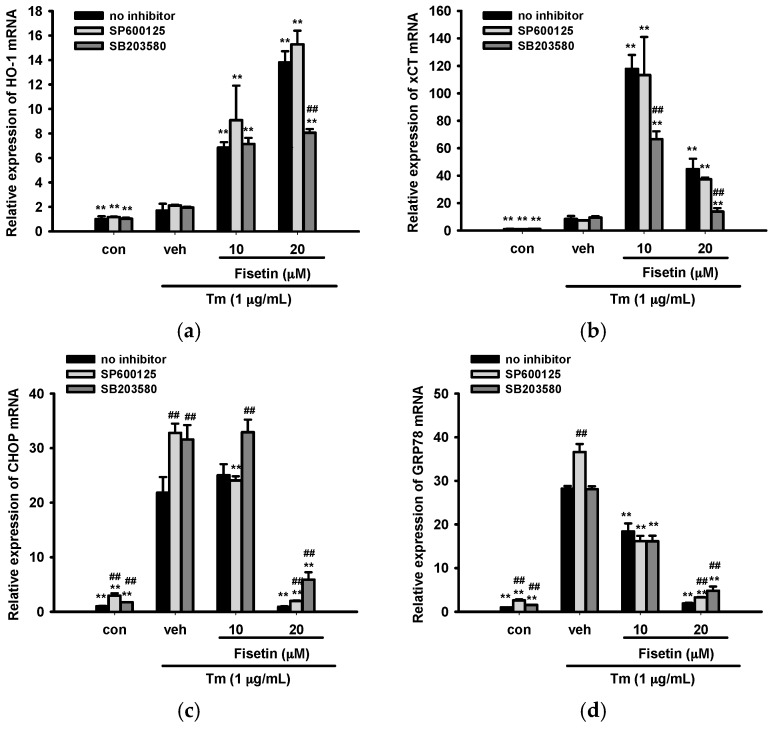
Effects of JNK and p38 MAPK inhibitors on UPR and oxidative stress response gene expression in response to Tm. PC12 cells were pretreated for 30 min with 5 µM JNK (SP600125) or p38 MAPK inhibitor (SB203580) and fisetin was then added 30 min prior to Tm exposure. (**a**,**b**) The mRNA levels of HO-1 and xCT were analyzed from RNA isolated after 12 h treatment of Tm; (**c**,**d**) The mRNA levels of CHOP and GRP78 were analyzed from RNA isolated after 6 h treatment of Tm. Data represent the mean ± SD of three independent experiments. ** *p* < 0.01 represents significant differences compared with the Tm-treated respective vehicle group. ## *p* < 0.01 represents significant differences compared with the respective no inhibitor group.

We further investigated whether JNK and p38 MAPK are involved in fisetin-mediated oxidative stress response gene expression. Figure 8a,b show that SB203580 significantly attenuated fisetin-enhanced HO-1 and xCT expression. This implies that fisetin-activated p38 MAPK signaling may be involved in upregulation of Nrf2-driven gene expression.

Furthermore, we investigated the role of JNK and p38 MAPK on ER stress responsive gene expression. Figure 8c,d show that Tm-mediated CHOP expression was increased by both SP600125 and SB203580, but GRP78 was only enhanced by SP600125. Furthermore, the 20 µM fisetin-inhibited expressions of CHOP and GRP78 were reversed by both SP600125 and SB203580. This indicates that both fisetin-mediated JNK and p38 MAPK activation may participate in down-regulation of ER stress. This result is in agreement with a previous finding that Tm-induced p38 MAPK activation may serve as an upstream negative regulator of ER stress, and confer adaptive cytoprotection against Tm-mediated cell injury [35].

### 2.8. Effect of Fisetin on Sirtuin 1 (SIRT1) Expression

**Figure 9:**
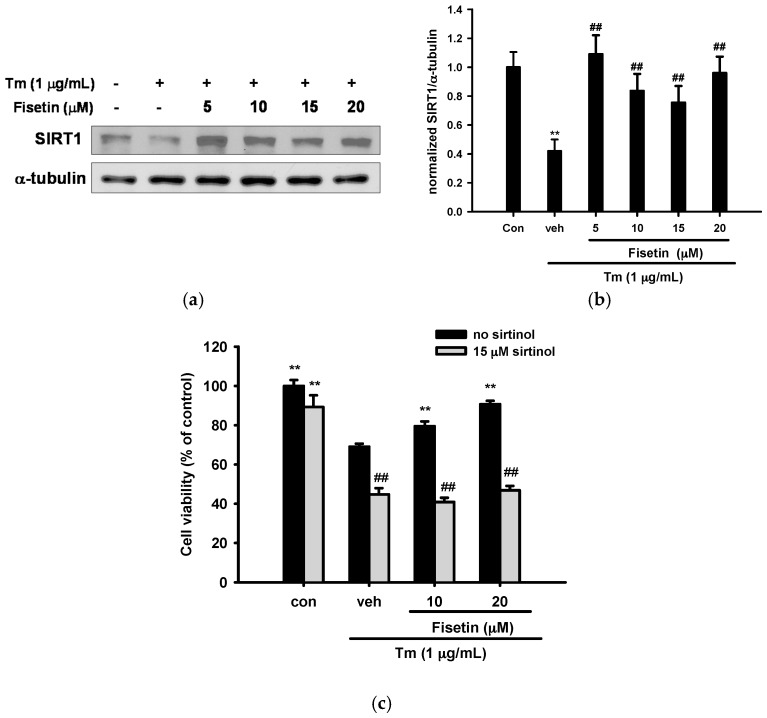
SIRT1 activity and overexpression is involved in the fisetin-mediated cytoprotective effect. (**a**,**b**) Fisetin reverses Tm-mediated SIRT1 downregulation. PC12 cells were treated with fisetin (5–20 µM) 30 min prior to Tm (1 µg/mL) treatment for 16 h at 37 °C. Cell lysates were prepared and the levels of SIRT1 and α-tubulin were analyzed by immunoblotting. Data represent the mean ± SD of three independent experiments. ** *p* < 0.01 represents significant differences compared with control. ## *p* < 0.01 represents significant differences compared with the Tm-treated vehicle group; (**c**) Effects of inhibition of SIRT1 activity on cell viability. PC12 cells were pretreated for 30 min with 15 µM sirtinol and 10 or 20 µM fisetin was then added 30 min prior to Tm (1 µg/mL) exposure for 16 h. MTT was used to analyze the cell viability. Data represent the mean ± SD of three independent experiments. ** *p* < 0.01 represents significant differences compared with the Tm-treated respective vehicle group. ## *p* < 0.01 represents significant differences compared with the respective no inhibitor group.

Fisetin is a SIRT1 activator that extends lifespan in lower organisms [44]. Fisetin also induces SIRT1 expression and inhibits early adipogenesis in 3T3-L1 cells [45]. We found here that Tm (1 µg/mL) treatment for 16 h significantly decreased SIRT1 expression, but co-treatment with fisetin (5–20 µM) reversed the reduction (Figure 9a,b).

To further examine the cytoprotective role of SIRT1, we examined whether the increased survival rate seen in fisetin-treated cells was dependent on SIRT1 activity. We pre-treated cells with sirtinol (SIRT1 activation inhibitor) for 30 min, followed by fisetin for 30 min before exposure to Tm (1 µg/mL) for 16 h. Figure 9c shows that the addition of sirtinol (15 µM) did not significantly cause cytotoxicity in PC12 cells in the absence of Tm. However, sirtinol completely blocked the fisetin-mediated cytoprotective effect against Tm. These results indicate that SIRT1 activity is involved in fisetin-induced cytoprotection.

## 3. Discussion

Phytochemicals are being studied for the therapeutic usage in the prevention and treatment of neurodegenerative disorders. Fisetin, a flavonoid existing in many different plant-based food products, has been reported to possess antioxidant, neurotrophic and neuroprotective activities by directly scavenging ROS or affecting signaling pathways in the maintenance of neuronal function and cell survival [25,26,27,28]. However, little is known regarding how fisetin affects signaling molecules in response to ER stress in neuronal cells.

We found here that Tm caused PC12 cell death. Addition of fisetin (5–20 µM) significantly reversed the cytotoxicity. We also tested whether fisetin could protect PC12 cells from another ER stress inducer, sesquiterpene lactone thapsigargin (Tg). It was found that Tg (0.3 µg/mL) caused 45% PC12 cell death and co-treatment with fisetin (5–20 µM) exerted a dose-dependent cytoprotective effect. The above results indicate that fisetin can prevent ER stress-induced cell death in PC12 cells (Figure S3). Furthermore, we also found that Tm (1 µg/mL) caused 33% adherent PC12 cell death, and only those cells supplemented with 15 and 20 µM fisetin could slightly attenuate the cytotoxicity, indicating that the cytoprotective effect of fisetin is valid to PC12 cells grown in both suspension and as monolayers on poly-l-lysine-coated plates (Figure S4).

Autophagy and apoptosis are cell death mechanisms with complex interactions between each other [46]. We found that fisetin inhibited Tm-mediated apoptosis, as indicated by decreased levels of activated PARP-1. Fisetin also decreased Tm-induced LC3-II accumulation and Atg12–Atg5 conjugate, indicating that Tm-mediated autophagy was also attenuated. Furthermore, our data show that in the absence of Tm, fisetin alone could protect PC12 cells from apoptotic damage. In contrast to the results observed for higher concentrations of fisetin, which induced apoptosis [30,31], this study is the first to document that low concentration of fisetin has anti-apoptotic and anti-autophagic effects in PC12 cells under ER stress.

To dissect the mechanism underlying the protective effect of fisetin, we first investigated whether fisetin inhibited Tm-induced ER stress gene expression. Our results show that fisetin did not inhibit XBP1 splicing or eIF2α phosphorylation. However, it significantly attenuated Tm-mediated mRNA expressions of GRP78, CHOP and TRB3. Significant inhibition against GRP78 protein overexpression was also noted. These results suggest that fisetin down-regulated ER stress-target gene expression without directly affecting Tm-activated IRE or PERK signaling.

It has been reported that high concentration of fisetin induces ER stress gene expression in cancer cells [30,32]. We therefore investigate whether low concentration of fisetin alone could upregulate unfolded protein response (UPR) gene expression in order to conquer ER stress. Figure S5a shows that no XBP1s RNA could be induced by fisetin (5–20 µM) after 6 h treatment. It has been reported that fisetin (<10 µM) dose- and time-dependently induces ATF4 expression in immortalized mouse hippocampal HT22 cells [41]. In concert with this, we found that treatment of PC12 cells with fisetin (5–20 µM) alone for 6 h caused modest stimulation with regard to mRNA expression of ATF4, GRP78 and CHOP following a hormetic dose–response curve, and the highest induction was found at 10–15 µM by 1.5- to 2.5-fold (Figure S5b–d). This suggests that the modest induction of UPR by fisetin alone may play a part in protecting cells from Tm-mediated ER stress.

It was reported that Tm induced ROS production in vitro and in vivo [40,47]. In the current study, we found that both fisetin and NAC, a synthetic precursor of GSH, can block Tm-mediated ROS production, as measured by the reduced DCF fluorescence. However, ROS are difficult to measure and prone to artifacts that can generate false-positive signals [48]. Many inherent problems, such as being oxidized by other reactive molecules, exist in using fluorescence probe H_2_DCFH to analyze H_2_O_2_ levels [49]. In addition, studies using antioxidants to demonstrate involvement of ROS [50,51] are not always conclusive. However, until newer ROS-detection techniques are evolved, a clear picture of Tm-induced ROS in PC12 cells will be lacking.

Nrf2 and ATF4 are critical transcription factors involved in GSH metabolism [52,53]. Therefore, compounds that upregulate both Nrf2 and ATF4 are potentially useful in neuroprotection through their effects on GSH metabolism. It has been reported that fisetin concurrently regulates Nrf2- and ATF4-driven gene expression, and increases GSH levels at both normal and under oxidative stress conditions in HT22 cells [41]. In accordance with this, we found that in addition to the abovementioned ATF4 induction, fisetin alone also stimulates Nrf-2-driven mRNA expression of Phase II antioxidant enzymes, HO-1, GCL and xCT in a hormetic effect.

Tm alone only weakly or insignificantly induced HO-1, GCLC and GCLM expression. On the other hand, xCT was stimulated by Tm alone more than 10-fold, supporting the notion that it was upregulated by both ATF4 and Nrf2. Fisetin treatment further enhanced HO-1 expression dose-dependently, and the cytoprotective role of HO-1 was confirmed by the addition of competitive inhibitor, Znpp. Fisetin also upregulated the genes involved in GSH metabolism in the presence of Tm. In conclusion, fisetin may exert a cytoprotective effect by increasing HO-1 expression and maintaining the GSH level via upregulating GCL and xCT expression [25].

We also found that fisetin activated ERK, JNK and p38 MAPK, and attenuation of JNK and p38 MAPK phosphorylation reduced its cytoprotective effects. Many studies reveal that the upregulation of HO-1 requires p38 MAPK activation [35,54,55,56]. In agreement with this, we found that SB203580 (p38 inhibitor), but not SP600125 (JNK inhibitor), attenuated fisetin-mediated HO-1 overexpression. It was also reported that p38 MAPK mediates cell survival in response to oxidative stress via induction of other Phase II antioxidant enzymes [57]. Inhibition of fisetin-activated p38 MAPK also strongly enhanced expression of ER stress-induced apoptotic CHOP, and a less prominent effect was found for those cells treated with JNK inhibitor. The above results suggest that fisetin-induced p38 MAPK phosphorylation, and to a lesser extent, JNK phosphorylation, may both confer adaptive responses to resist Tm-mediated ER stress and cytotoxicity.

The development of sirtuin-activating compounds (STACs) as nutraceuticals in the management of chronic diseases has attracted considerable research interest in recent years, with fisetin found to be a SIRT1 activator and inducer [44,45]. We found that fisetin reversed Tm-inhibited SIRT1 expression in PC12 cells. In the presence of SIRT1 inhibitor sirtinol, no fisetin-mediated cytoprotective effect could be observed. These results indicate that fisetin suppresses neuronal toxicity, possibly via modulating SIRT1 activation and expression.

## 4. Materials and Methods

### 4.1. Materials

Fisetin (≥98%), tunicamycin (Tm), MTT (3-(4,5-dimethylthiazol-2-yl)-2,5-diphenyltetrazolium bromide), RPMI-1640 medium, and other chemicals were from Sigma-Aldrich Co. (St. Louis, MO, USA), unless otherwise indicated. 1,4-Diamino-2,3-dicyano-1,4-bis(o-aminophenylmercapto) butadiene (U0126), a selective and potent inhibitor of MEK activity and activation of ERK1/2, and 4-(4′-fluorophenyl)-2-(4′-methylsulfinylphenyl)-5-(4′-pyridyl)-imidazole (SB 203580), a p38 MAP kinase inhibitor, were purchased from Promega (Madison, WI, USA). SP600125 (1,9-pyrazoloanthro**n**), an inhibitor of JNK, was from Calbiochem (San Diego, CA, USA). Sirtinol, a specific inhibitor of SIRT1 and SIRT2, was from Santa Cruz (Dallas, TX, USA).

### 4.2. PC12 Cell Culture

The rat adrenal pheochromocytoma cell line PC12 was obtained from the Bioresource Collection and Research Center (Hsinchu, Taiwan) and maintained in RPMI-1640 medium, which contains 2 mM glutamine, 1.5 g/L sodium bicarbonate, 4.5 g/L glucose, 10 mM HEPES, 1 mM sodium pyruvate, 100 U/mL penicillin and streptomycin, supplemented with 10% heat-inactivated horse serum (Hyclone, Logan, UT, USA) and 5% fetal bovine serum (Invitrogen, Carlsbad, CA, USA) in 5% CO_2_ incubator at 37 °C.

### 4.3. Drug Treatments and Cell Viability Assay

For experiments testing the ability of tunicamycin (Tm) to induce cytotoxicity, cells were incubated in serum-free RPMI medium in the presence of Tm [34]. PC12 cells (5 × 10^5^/mL) were seeded in 24-well plates and pretreated with the indicated concentration of fisetin or an equivalent volume of DMSO vehicle control (final concentration of 0.1%) for 30 min, followed by Tm treatment for 16 h.

To measure the cell viability of adherent PC12 cells that had undergone Tm-induced damage, PC12 cells (5 × 10^5^/mL) were seeded on poly-l-lysine-coated 6-well plates in low serum (0.5% fetal bovine serum and 1% horse serum) medium for 16 h. Fisetin or an equivalent volume of DMSO vehicle (final concentration of 0.1%) was then added and incubated for 30 min prior to Tm treatment for an additional 16 h.

A cell-free blank with medium and tested reagent was employed in parallel. Cell viability was assessed by the mitochondrial-dependent reduction of MTT to purple formazan [58]. The cell viability was calculated by subtracting the OD_550_ of cell-free blank from OD_550_ of each sample and was expressed as percentage of the control (100%).

An esterase-dependent cell viability analysis Calcein AM (Invitrogen) was used for reconfirmation of MTT data [59]. Briefly, cells were incubated with 5 µM Calcein AM for 30 min at 37 °C, and the fluorescent signal was monitored using 485 nm excitation and 530 nm emission wavelengths.

### 4.4. Protein Extraction and Immunoblotting

RIPA buffer (Thermo Fisher Scientific, Inc., Rockford, IL, USA) was used for preparation of whole cell extracts and the protein concentration was measured by the Bradford method (Bio-Rad Laboratories, Hercules, CA, USA). Equal amounts of cell lysates were separated on SDS-PAGE and then transferred onto Hybond-P PVDF (GE Healthcare, Buckinghamshire, UK) using a CAPS transfer buffer at 30 mA overnight at 4 °C. The membranes were blocked in a freshly made blocking buffer (5% skim milk in PBS with 0.05% Tween 20, pH 7.4, PBS-T) for 6 h at room temperature. After washing with PBS-T, the membranes were incubated with an appropriate dilution (1:1000‒1:5000) of primary antibody (Table 1) overnight at 4 °C on a rocking platform. The membranes were then washed and incubated with suitable horseradish peroxidase-conjugated secondary antibody (Jackson ImmunoResearch, West Grove, PA, USA, at a dilution of 1:10,000‒1:25,000) for 1 h at room temperature. The blots of were incubated by ECL Prime (GE Healthcare), and the chemiluminescent signals were then visualized with X-ray film. Densitometry of the bands was analyzed by ImageJ software version 1.50 (National Institutes of Health, Bethesda, MD, USA).

### 4.5. RNA Extraction, Real-Time RT-PCR, and Semi-Quantitative RT-PCR

Illustra RNAspin Mini RNA Isolation Kit (GE Healthcare) was used for preparation of total RNA. High-Capacity cDNA Archive Kit (Applied Biosystems, Waltham, MA, USA) was used to prepare cDNA from 1 µg RNA. Real-time PCR (StepOne Real-Time PCR System, Applied Biosystems) was performed with 2 µL of the cDNA, 200 nM primers (Table 2) and Fast SYBR Green Master Mix (Applied Biosystems) in 25 µL reaction mixture. The amplification conditions were as follows: 95 °C for 2 min, 40 cycles at 94 °C for 15 s, and 60 °C for 60 s. Target gene expression was measured and normalized to the respective β-actin expression level. The identity and purity of the amplified product was checked through analysis of the melting curve carried out at the end of amplification. Relative expression was evaluated with the ΔΔ*C*_t_ method.

XBP1s and XBP1u mRNA levels were measured using regular PCR as described in our previous publication [60].

### 4.6. Intracellular ROS Analysis

Cellular reactive oxygen species were analyzed using the fluorescence probe 2′,7′-dichlorodihydrofluorescein diacetate (H_2_DCFDA) (Invitrogen), which passively diffuses into the cell and is cleaved and oxidized to 2′,7′-dichlorofluorescein (DCF). PC12 cells were stained with 20 µM H_2_DCFDA at 37 °C for 30 min in the dark and then washed once in PBS. Fluorescence dye loaded cells were seeded in black 96-well plates (2 × 10^5^/well) and treated with fisetin or in combination with Tm for 16 h. The signals were then read at EX485 nm/Em535 nm using a fluorometer.

### 4.7. Statistical Analysis

All experiments were repeated at least three times. The results were analyzed using Kruskal–Wallis *H* Test by SPSS version 18. If the Kruskal–Wallis *H* Test shows a significant difference between the groups, then pairwise comparisons were employed by Mann–Whitney *U* Tests, and a *p*-value of <0.05 was taken to be significant.

## 5. Conclusions

Fisetin (5–20 µM) protected PC12 cells from Tm-induced cell death. Fisetin mitigated Tm-mediated apoptosis, autophagy and reactive oxygen species (ROS) production. Its cytoprotective effects were likely associated with modulation of UPR and resulted in down-regulation of ER stress target genes, GRP78, CHOP, and TRB3. Furthermore, treatment of cells with fisetin induced expression of Nrf2 target genes, HO-1, GCLC, GCLM and xCT. Fisetin restored SIRT1 expression and enhanced MAPK activation, which may confer an adaptive response to modulate Tm-mediated stress responses and cytotoxicity.

## Figures and Tables

**Table 1:** Primary antibodies used in Western blotting.

Antibody	Company	Catalog Number
α-tubulin	Sigma	T 6199
β-actin	GeneTex (Hsinchu, Taiwan)	GTX629630
PARP-1	Santa Cruz	H-250
LC3B	Gene Tex	GTX127375
Atg12	Gene Tex	GTX124181
eIF2α	Gene Tex	GTX101241
*p*-eIF2α	Gene Tex	GTX61039
GRP78	BD (Franklin Lakes, NJ, USA)	610978
CHOP	Santa Cruz	sc-7351
HO-1	Enzo Life Sciences (Farmingdale, NY, USA)	SPA-895
ERK	Cell Signaling (Danvers, MA, USA)	4695
*p*-ERK	Cell Signaling	4370
p38	Cell Signaling	9212
*p*-p38	Cell Signaling	9215
JNK	Cell Signaling	9258
*p*-JNK	Cell Signaling	4668
SIRT1	Cell Signaling	8469

**Table 2:** Primer pairs used in RT-Q-PCR.

Gene	Primers	Amplicon (bp)
β-actin [61]	(F) CCTCTGAACCCTAAGGCCAA (R) AGCCTGGATGGCTACGTACA	90
ATF4 [39]	(F) CTTCTCCAGGTGTTCCTCGT (R) TGCTCAGCCCTCTTCTTCTG	163
CHOP [35]	(F) AAGAATCAAAAACCTTCACTACTCTTGACC (R) TGGGAGGTGCTTGTGACCTCTGC	91
GCLC [62]	(F) TGGCCAGCCGTACGGAGGAA (R) CAGGGAGCCTAGCCTGGGA	143
GCLM [62]	(F) CTTTCCTTGGAGCATTTGCAGCCTT (R[35]) ACCTGTGCCCACTGGTACAGCTG	131
GRP78 [35]	(F) CAACTCACGTCCAACCCGGAGAA (R) TGTCTTGGTTTGCCCACCTCCG	171
HO-1 [63]	(F) GCCTGCTAGCCTGGTTCAAG (R) AGCGGTGTCTGGGATGAACTA	87
TRB3 [39]	(F) GGACAAGATGCGAGCCACAT (R) CCACAGCAGGTGACAAGTCT	179
xCT [60]	(F) GACAGTGTGTGCATCCCCTT (R) GCATGCATTTCTTGCACAGTTC	110

## Supplementary Materials

Supplementary materials can be found at www.mdpi.com/1422-0067/18/4/852/s1.
