# Supplementary material for: Fisetin Protects PC12 Cells from Tunicamycin-Mediated Cell Death via Reactive Oxygen Species Scavenging and Modulation of Nrf2-Driven Gene Expression, SIRT1 and MAPK Signaling in PC12 Cells
Source: Int J Mol Sci. 2017 Apr 17;18(4):852. doi: 10.3390/ijms18040852 (PMC5412436; doi:10.3390/ijms18040852)
Supplement: Supplementary file 1 [file ijms-18-00852-s001.pdf]

Supplemental Figures

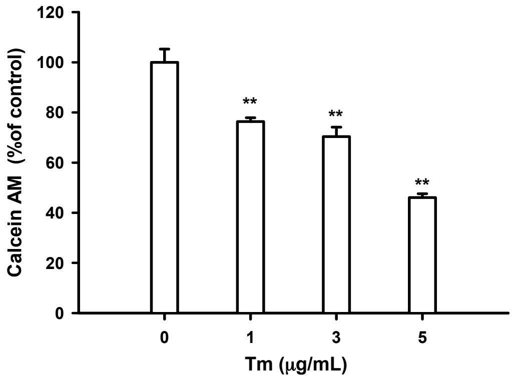

(**a**)

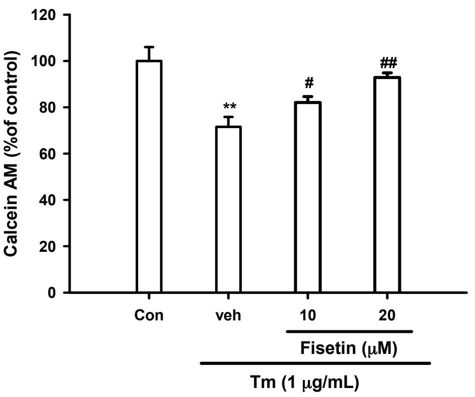

(**b**)

Figure S1. (**a**) Tm causes cell death in PC12 cells; (**b**) Fisetin inhibits Tm-mediated cytotoxicity in PC12 cells. Cells were treated with the indicated concentration of compound or vehicle control (0.1% dimethyl sulfoxide, DMSO) for 30 min followed by exposure to Tm for an additional 16 h at 37 °C. Cell viability was measured by Calcein AM dye staining, as described in Materials and Methods. **, $p < 0.01$ represents significant differences compared with vehicle control (Con, without Tm). #, p<0.05; ##, $p < 0.01$ represents significant differences compared with the Tm-treated vehicle (veh).

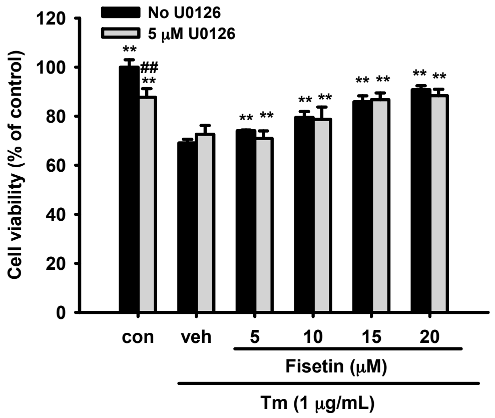

Figure S2. Effect of inhibition of ERK activation on cell viability. PC12 cells were pretreated for 30 min with 5 μM ERK inhibitor (U0126) and 5–20 μM fisetin was then added 30 min prior to Tm (1 μg/mL) exposure for 16 h. MTT was used to analyze the cell viability. Data represent the mean ± SD of three independent experiments. ** $p < 0.01$ represents significant differences compared with the Tm-treated respective vehicle group. ##, $p < 0.01$ represent significant differences compared with the respective no inhibitor group.

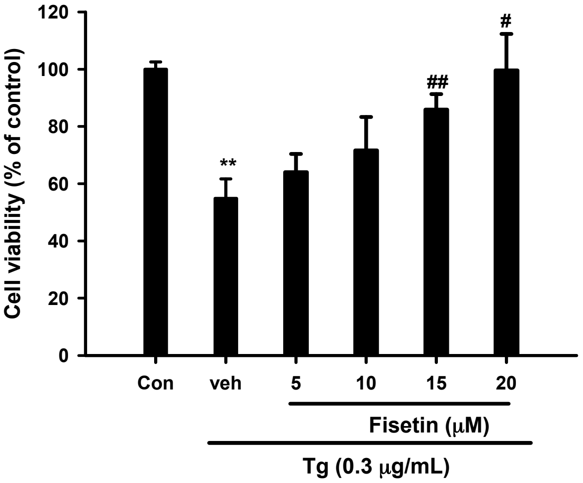

Figure S3. Effect of fisetin on Tg-mediated cytotoxicity in PC12 cells. Cells were treated with the indicated concentration of compound or vehicle control (0.1% DMSO) for 30 min followed by exposure to Tg for an additional 16 h at 37°C. Cell viability was measured by MTT, as described in Materials and Methods. **, p<0.01 represents significant differences compared with vehicle control (without Tm). #, p<0.05, and ##, p<0.01 represent significant differences compared with the Tm-treated vehicle group.

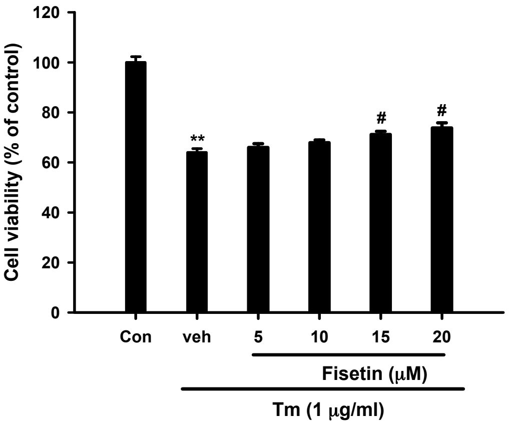

Figure S4. Effect of fisetin on Tm-mediated cytotoxicity in adherent PC12 cells. PC12 cells were cultured on poly-L-lysine-coated plates in RPMI-1640 supplemented with 0.5 % fetal bovine serum and 1% horse serum (low serum) for 16 h prior to the addition of indicated reagent for 30 min followed by Tm treatment for additional 16 h. Cell viability was measured by MTT as described in Materials and Methods. Data represent the mean ± SD of three independent experiments. **, p<0.01 represents significant differences compared with vehicle control (without Tm). #, p<0.05 represents significant differences compared with Tm-treated vehicle group.

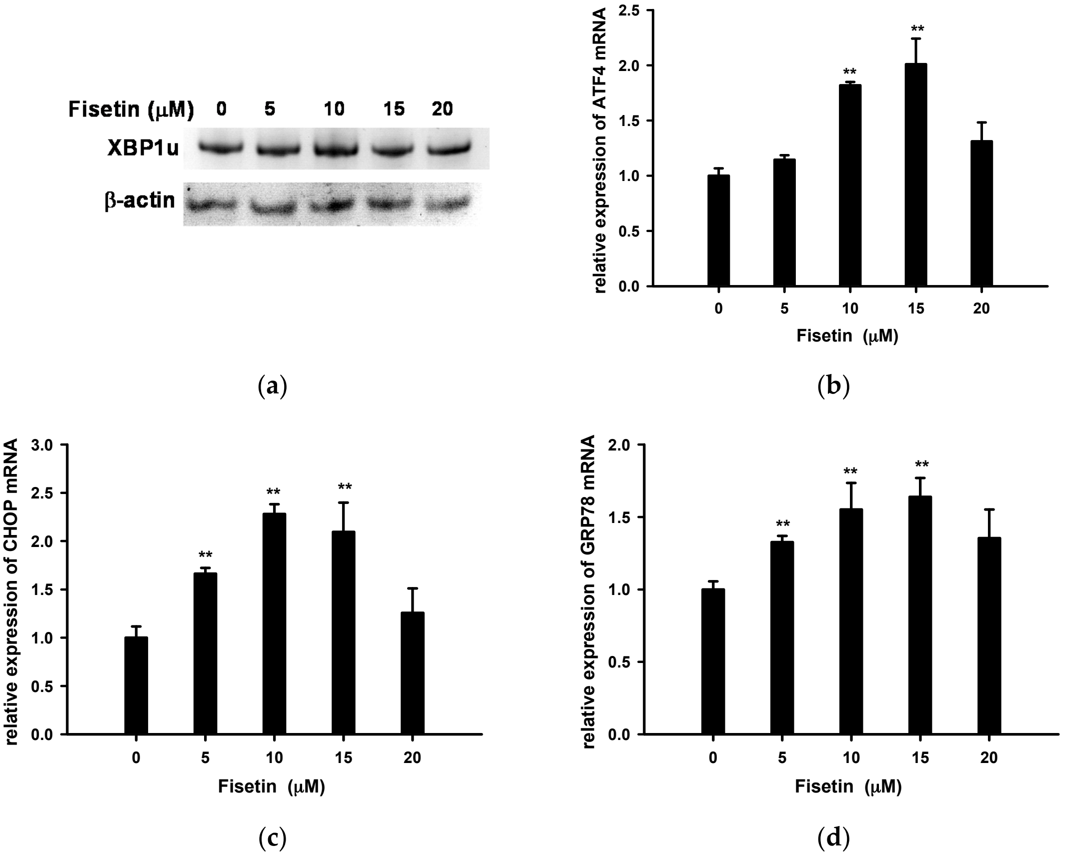

Figure S5. Effect of fisetin alone on unfolded protein response. PC12 cells were treated with fisetin for 6 h at 37°C and RNA was prepared. Semi-quantitative RT-PCR was used for the analysis of mRNA levels of XBP1s, XBP1u and β-actin. RT-Q-PCR was used for the analysis of mRNA levels of ATF4, GRP78, CHOP and TRB.
